# Totally implantable venous ports in infants and children: a single-center retrospective study of indications and safety

**DOI:** 10.3389/fonc.2024.1351630

**Published:** 2024-04-16

**Authors:** Patrycja Sosnowska-Sienkiewicz, Sebastian Moryciński, Danuta Januszkiewicz-Lewandowska, Karolina Michalik, Klaudyna Madziar, Agata Kukfisz, Daria Zielińska, Przemysław Mańkowski

**Affiliations:** ^1^ Department of Pediatric Surgery, Traumatology and Urology, Poznan University of Medical Sciences, Poznan, Poland; ^2^ Department of Pediatric Oncology, Hematology and Transplantology, Poznan University of Medical Sciences, Poznan, Poland; ^3^ Department of Perinatology and Gynecology, Poznan University of Medical Sciences, Poznan, Poland; ^4^ Faculty of Medical Sciences in Zabrze, Medical University of Silesia, Zabrze, Poland; ^5^ Provincial Hospital for Neurological and Mental Illness, Lubiaz, Poland

**Keywords:** long-term access, child, Seldinger method, venous port, complications, infections

## Abstract

**Introduction:**

Totally Implantable Venous Access Devices (TIVADs) contribute significantly to the treatment progress and comfort of patients requiring long-term therapy. However, the procedure for implanting TIVADs, as well as its very presence, may be associated with complications.

**Aim:**

This study evaluates the indications, safety, and complication rates of venous port implantations in pediatric patients. It also explores factors influencing the occurrence of early and late complications post-implantation.

**Materials and methods:**

The study included 383 pediatric patients treated at the Department of Pediatric Surgery, Traumatology, and Urology in Poznan between 2013 and 2020 who underwent 474 implantations of intravenous ports. Venous access was achieved using the Seldinger technique. Statistical analysis was performed using Statistica 13 with TIBCO and PQStat 1.8.2.156 with PQStat.

**Results:**

Venous ports were used in 345 oncology patients requiring chemotherapy (90% of the total group) and in 38 children (10%) with non-oncology indications. There were 36 early complications (7.6%) and 18 late complications (3.8%), excluding infectious complications. The most common early, non-infectious complications included pneumothorax (15 patients; 3%) and port pocket hematoma (12 patients; 2.5%). The most common late, non-infectious complications observed were venous catheter obstruction (8 children; 1.7%) and port system leakage (5 children; 1%). Infectious complications occurred in 129 cases (27.2%). Children with a diagnosis of non-Hodgkin’s lymphoma, acute myeloid leukemia, and acute lymphoblastic leukemia had a significantly higher incidence of port infections. Venous ports equipped with a polyurethane catheter, compared to systems with a silicone catheter, functioned significantly shorter.

**Conclusions:**

The Seldinger method of port implantation is quick, minimally invasive, and safe. The type of port, including the material of the port’s venous catheter, and the underlying disease have an impact on the durability of implantable intravenous systems. The experience of the surgeon is related to the frequency of complications associated with the procedure.

## Introduction

Totally Implantable Venous Access Devices (TIVADs), commonly referred to as ports or vascuports, are subcutaneously implanted systems for long-term treatment. The indications for implantation of TIVADs are very broad. They play an important role in providing long-term venous access. In the pediatric group, indications include the administration of chemotherapy, and long-term intravenous antibiotic therapy. They are also used in chronic disease management and total parenteral nutrition (1–7). The subcutaneous position of the system allows patients to function comfortably without the risk of catheter damage. Children can participate in sports activities, including swimming (1, 2, 8). The venous port consists of a capsule connected to a central venous catheter (2). In the central part of the port is a silicone cured membrane, and beyond it - the port chamber. To access the chamber, the membrane is punctured percutaneously with a specially designed non-cutting Huber needle (2, 9).

The optimal location for the venous port capsule is the fascia of the pectoralis major muscle, on the anterior surface of the chest, in the subclavian region. A change in the standard location of the venous port may be dictated by conditions that prevent implantation in the subclavian region. The location of the port is determined by the possible intravenous access (subclavian or femoral vein) (10, 11). Venous ports in children are placed under general anesthesia.

Depending on the literature reviewed, variable rates of complications after port implantation are described. They are typical of the complications of central venous cannulation (12, 13). Early complications associated with port implantation by subclavian vein cannulation include pneumothorax, pleural hematoma, cardiac arrhythmia, puncture of the subclavian artery, displacement of the venous catheter, bleeding into the port locus, or early infection.

The most common late complications include infection, port obstruction, system leakage, dermatitis, catheter stenosis associated with compression in the subclavian space between the clavicle and first rib, thrombosis associated with the presence of the catheter in the vessel, and displacement of the catheter tip. It is suggested that factors that increase the risk of infection include the type of chronic disease (acute lymphoblastic leukemia, acute leukemia non-lymphoblastic), low absolute neutrophil count, patient malnutrition, fever on the day of surgery, chemotherapy initiated, and ongoing steroid therapy (1, 14–17).

The primary aim of this study was to evaluate the safety of the venous port implantation technique and to identify the most common early and late post-implantation complications.

In addition, the study aims to dissect the variables influencing the incidence of these complications and to delineate those attributable to patient factors and procedural specifics.

## Materials and methods

### Patients and study design

From 2013 to 2020, 383 patients underwent TIVAD implantation at the Department of Pediatric Surgery, Traumatology, and Urology in Poznan. Retrospective analysis of clinical data of patients was performed.

The medical records of the patients were analyzed in terms of the implantation procedure, the type of port, the experience of the surgeon performing the procedure, and the occurrence of early and late complications.

Only those patients whose medical records were confirmed to be complete from the time of venous port implantation to the day of removal were analyzed.

Laboratory parameters constituting absolute contraindications to venous port implantation were Hb levels below 8g%, platelet counts below 40,000/mm^3, and INR values above 1.5.

Complications related to port implantation were divided into early complications, directly related to the surgical procedure, observed up to 30 days after the procedure, and late complications, related to the operation of the port, observed more than 30 days after the operation. Parameters assessed as likely to affect venous port implantation and function were divided into patient-dependent (age at implantation, gender, weight, clinical diagnosis, laboratory results) and operator-dependent (surgeon experience - resident vs specialist, type of venous access, duration of surgery, type of implanted port) factors.

The study was approved by the Bioethics Committee at the Poznan University of Medical Sciences.

### Operation technique and postoperative standards

All port implantation procedures were performed in an operating room setting, with aseptic principles, under general anesthesia. Venous access was obtained using the Seldinger technique. Before the start of the procedure, the appropriate size of the port was chosen adapted to the patient’s weight and anatomical conditions. Two types of low-profile venous ports were used: 8.7 mm high port equipped with a 4.5 F diameter polyurethane catheter for younger children and 10 mm high port equipped with a 6.6 F diameter silicone catheter for older children.

Standard postoperative care included continuous monitoring of vital signs and blood counts. Prior to 2018, a follow-up chest X-ray in a-p projection was routinely performed 3-4 hours after the procedure. However, post-2018 protocols were updated to require chest X-ray imaging only under suspicion of emphysema or bleeding, relying on close nursing and medical supervision for monitoring vital functions.

### Statistical analysis

Statistical analysis was performed using Statistica 13 from TIBCO and PQStat 1.8.2.156 from PQStat. α=0.05 was used as the level of significance. A result was considered statistically significant when p<α. For the analysis of factors causing the need for early port removal or infectious complications for categorical variables, the chi2 test of independence, Fisher’s exact test, or Fisher-Freeman-Halton test was calculated. If a relationship was found, an odds ratio was calculated along with a 95% confidence interval. The normality of the distribution of continuous variables was tested with the Shapiro-Wilk test. For continuous variables, such as age at implantation, body weight, and laboratory parameters, the Mann-Whitney test was used due to the lack of conformity to a normal distribution. In addition, a Kaplan-Meier reliability analysis was performed to determine port durability. The LogRank test was used to compare port durability between groups. The Cox proportional hazards model was also used to determine the risk ratio (Hazard ratio) of port loss along with 95% confidence intervals.

## Results

### Study group characteristics

In 383 patients, 474 central venous port implantation procedures were performed. The body weight of the operated children ranged from 3.7 kg (4-week-old newborn) to 95 kg (17-year-old female patient), with a mean of 28 kg with a median of 20 kg in the study group. The general characteristics of the study group are summarized in the table (**Table 1**).

**Table 1:** Characteristics of the study group.

Number of patients Female sex Male sex	383 167 (44%) 216 (56%)
Number of implantations Female sex Male sex	474 194 (41%) 280 (59%)
Age (range) Median (years) Average (years)	21 days-17 years 5 7
Follow-up of port functioning (range) Median (months) Mean (months)	2 months- 5 years 18 20

The most common indication for venous port placement was the need for long-term chemotherapy (345 children; 90.0%). This group of patients was dominated by children treated for acute lymphoblastic leukemia (131 patients; 34.2%), central nervous system tumors (56 patients; 14.6%), lymphomas (50 patients; 13.0%), and neuroblastoma (39 patients; 10.1%). A separate group of patients were non-oncology patients (38 patients; 9.9%). This group consisted of 11 (2.9%) children with the syndrome of congenital anomalies, 13 with hemophilia A (3.8%), 9 with immune disorders (2.3%), and 5 with cystic fibrosis (1.3%).

**Figure 1:**
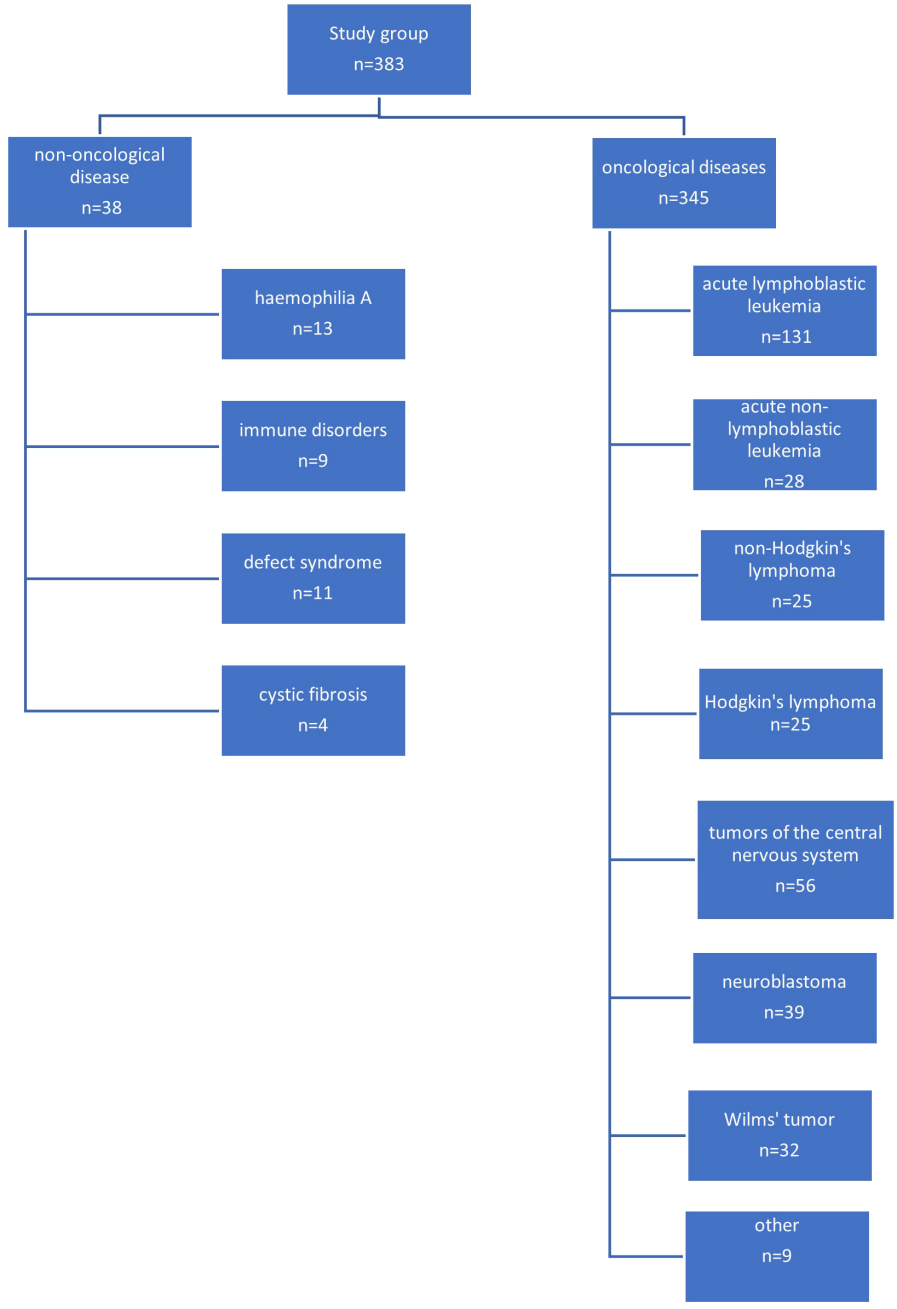
Diagram showing the final diagnoses of operated patients.

A diagram showing the specific indications for port implantation in the study group of children is shown in **Figure 1**.

### Surgical treatment

In all of the patients, venous access was achieved using the Seldinger method. During 332 (70.0%) implantations, the catheter was inserted through the right subclavian vein, during another 140 (29.5%) implantations through the left subclavian vein, in 2 children, the right femoral vein was cannulated. Venesection, surgical dissection, and exposure of the vein were not used as a method of accessing the vessel.

Depending on the patient’s clinical condition and the results of laboratory tests, 173 (36.4%) implantations required the administration of blood products before the port implantation procedure. Red blood cell concentrate was administered in 66 implantations (13.9%), platelet cell concentrate in 79 implantations (16.6%), plasma in 23 implantations (4.9%), and plasma clotting factor concentrate in 5 implantations (1.0%).

The average procedure time for port implantation was 38.5 minutes +/- 16.6 minutes. The shortest procedure took 10 minutes, and the longest 155 minutes. Half of all procedures performed lasted at most 35 minutes, and 1/4 lasted at least 45 minutes. The duration of the procedure depended on the experience of the operator (p=0.016). The mean duration of venous port function was 606 days (1 year and 8 months), with a median of 544 days (1.5 years).

### Complications

In a group of 38 non-oncology patients, there were 3 early and 5 late complications. In 2 children with severe hemophilia, early postoperative complication was the formation of a giant hematoma of the operated area, and in 1 patient with immunodeficiency - pneumothorax. Of the late complications, 2 children with the syndrome of congenital anomalies developed catheter obstruction, and 3 children with immunodeficiency developed port infection.

Among the 345 oncology patients, there were 33 early complications (9.6%) and 13 late complications (3.8%) excluding infectious complications. Among early non-infectious complications, pneumothorax (14 patients) and hematoma (10 children) at the site of port implantation were the most common.

Pneumothorax as a complication of port implantation occurred more often when the operator was a resident physician in training (p= 0.02580). In 12 of 15 patients (80.0%) whose venous port insertion was complicated by pneumothorax formation, a pleural suction drain had to be placed.

Among late complications excluding infectious complications, we observed catheter obstruction in 6 children (1.7%), system leakage in 5 (1.0%), deep vein thrombosis in another 4 (0.8%), and catheter tip displacement in 1 child (0.2%).

### Infectious complications

**Figure 2:**
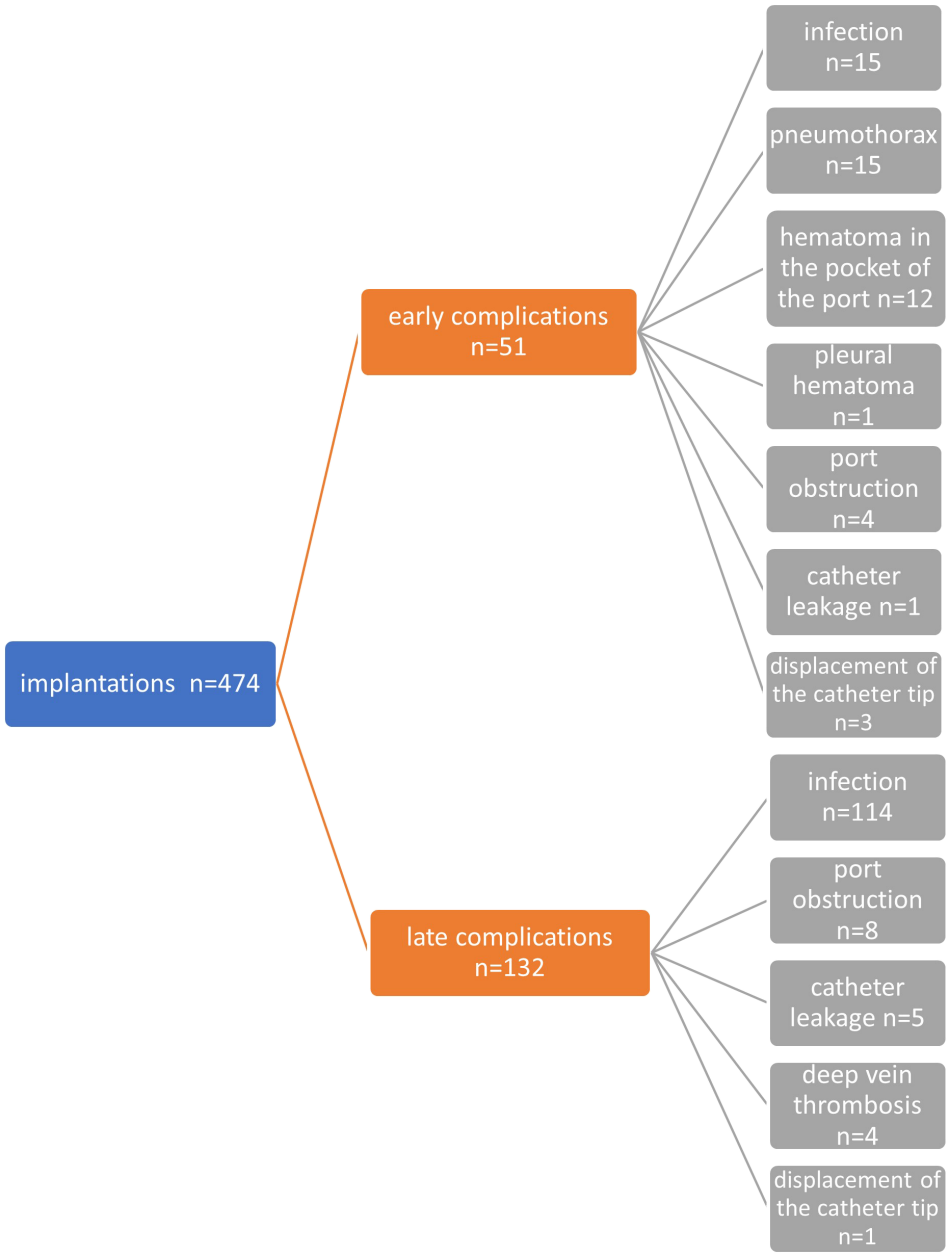
Diagram showing all early and late, non-infectious and infectious complications in the entire study group.

Infectious complications occurred in 129 cases of all implantations (27.2%). Early infectious complications up to 30 days after implantation that occurred in the oncology patient group were found in 12 cases (2.5%), and late infectious complications occurred in as many as 114 implantations (24.0%). A diagram showing the frequency and type of complications after venous port implantation is shown in **Figure 2**. There was no relationship between the duration of the procedure and the occurrence of venous port infection (p=0.413). There was no relationship between the patient’s CRP level at the time of venous port implantation and the occurrence of system infection (p=0.31639). There was also no significant effect of neutrophil (p=0.55759) and leukocyte (p=0.96035) levels at the time of port implantation on the occurrence of an infectious complication.

The study group of all oncology and non-oncology patients who received venous port implantation was divided into 4 age ranges: patients up to 1 year of age (n=48); patients between 1 and 5 years of age (n=166); patients between 5 and 10 years of age (n=107); and between 10 and 18 years of age (n=153). Early infections occurred in 2 (4.2%) patients under 1 year of age, 8 (4.8%) in the 1-5 year group, 3 (2.8%) children in the 5-10 year group, and 2 (1.3%) in the 10-18 year group. Late infections occurred in 17 (35.4%) patients under 1 year of age, 59 (35.5%) in the 1-5 year group, 23 (21.4%) children in the 5-10 year group, and 15 (9.8%) in the 10-18 year group. There was a significant difference in the rate of occurrence of infectious complications between the group of patients under 5 years of age and the other age groups. Significantly more infections occurred in the group of younger children (p=0.0035).

A correlation was shown between the diagnosis of the underlying disease and the rate of port infections. The highest rates of infections of implanted systems were associated with three specific diagnoses: non-Hodgkin’s lymphoma, acute leukemia, and acute lymphoblastic leukemia (p=0.00277, Cramer’s V correlation coefficient=0.246). The correlation between diagnoses and the incidence rate of venous system infections is shown in **Table 2**.

**Table 2:** The incidence of venous port infections according to the patient’s underlying disease.

Diagnosis	Number of patients (n)	Port infection (n; %)
Tumors of the central nervous system *	56	14 (25%)
Wilms' tumor *	32	1 (3.125%)
Neuroblastoma *	39	15 (38.5%)
Hodgkin's lymphoma *	25	5 (20%)
Non-Hodgkin's lymphoma *	25	15 (60%)
Acute non-lymphoblastic leukemia	28	15 (67.9%)
Acute lymphoblastic leukemia	131	55 (41.98%)
Other	47	9 (19%)
Total **	**474**	**129 (27%)**

*Tumors and lymphomas requiring treatment with chemotherapy and/or radiation therapy.

**Selected patients had multiple port implantations.

**Figure 3:**
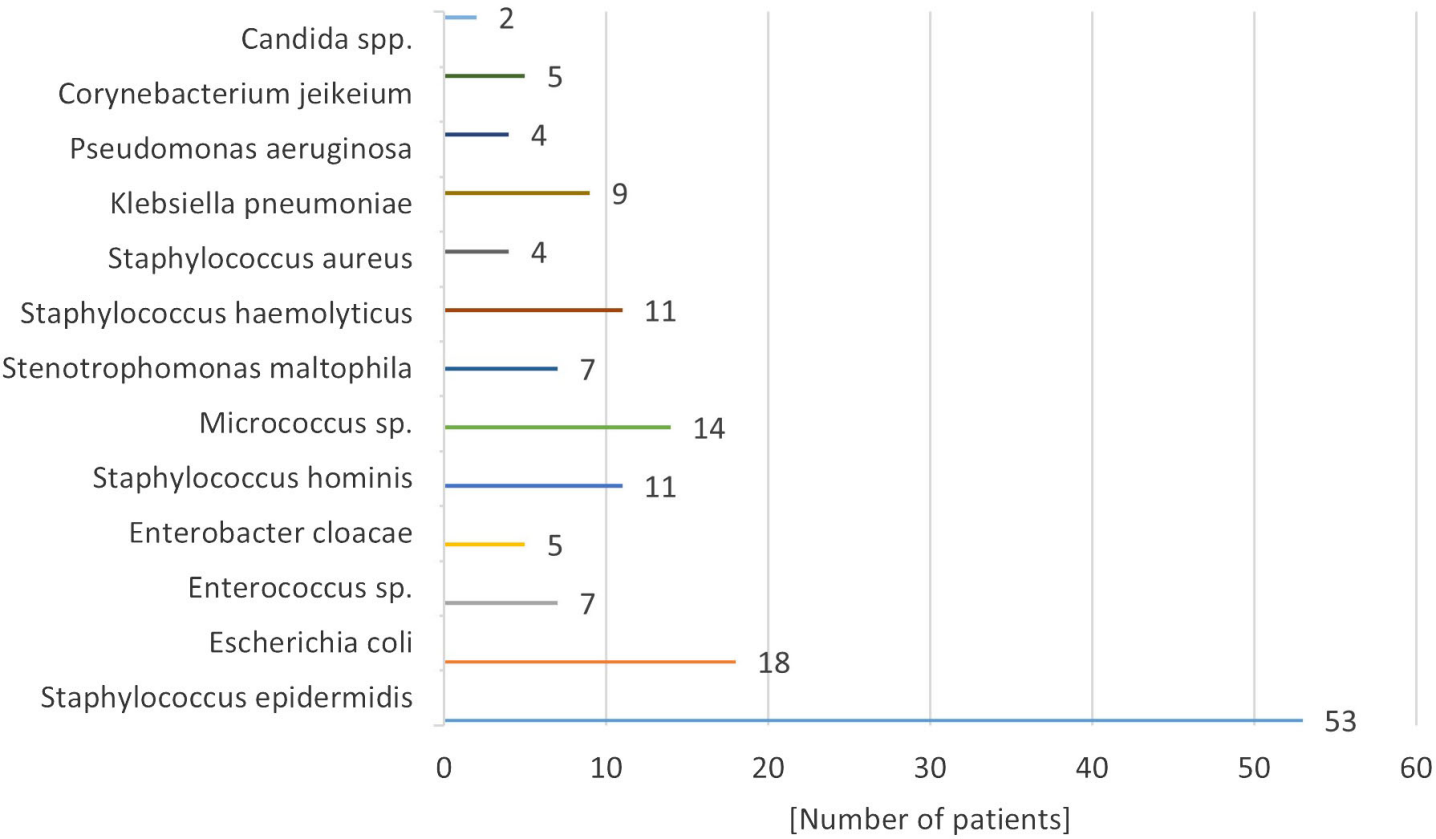
Bacteriological results obtained from the patient’s blood culture taken from the port.

Microbiological results indicated that the predominant microorganisms infecting implantable venous accesses were skinborne bacteria, including gram-positive granulomas and gram-negative bacilli. The specific bacterial pathogen causing fulminant septicemia was *Staphylococcus haemolyticus* (11.0% of infected systems) (**Figure 3**). *Candida* spp. fungal infection was confirmed in 2 patients. ---

In 129 cases of early and late infectious complications of the venous port, 96 cases were treated with antibiotic therapy alone as the primary treatment for the infection. In this group, in 35 cases, the port was removed after 2-8 days despite antibiotic therapy. In 33 cases, in addition to antibiotic therapy, the venous port was removed urgently on the first day of the infection. In total, removal of the system was necessary in 68 patients (53.0% of patients with an infectious complication). In the entire study group 14.0% of children lost their port due to infectious complications. The introduction of targeted antibiotic therapy and conservative treatment of venous port infection was effective in 61 children (47.2% of all infected systems). The average duration of antibiotic therapy was 14 days, ranging from 7 to 23 days. In the presence of coagulase-negative and methicillin-resistant *staphylococci*, vancomycin and teicoplanin were used, while piperacillin with tazobactam, aminoglycosides, and carbapenems were used to eradicate gram-negative bacilli. When fungal infection was confirmed (in 2 children) within the implanted venous system, the port was immediately removed in each case, in addition to the intravenous antifungal treatment used.

### Length of implanted venous system function

**Figure 4:**
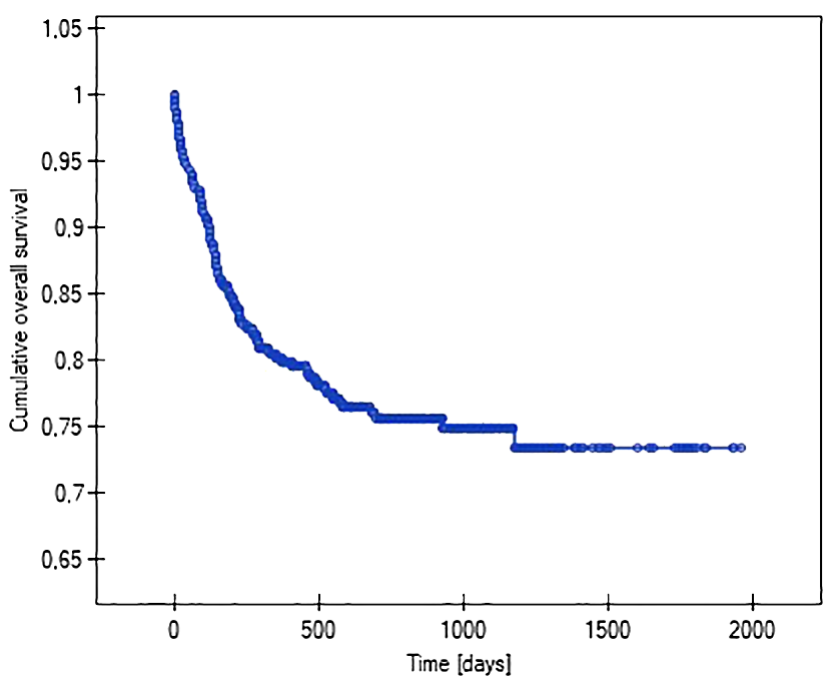
Graph showing the analysis of the predicted length of vascular port function.

Using Kaplan-Meier analysis, the lengths of the function of venous systems implanted in patients with various malignancy diagnoses were evaluated (i.e., so-called survival analysis). It showed that patients with acute myeloid leukemia (47%), non-Hodgkin’s lymphoma (37%), and acute lymphoblastic leukemia (28%) had the highest probability of losing their venous systems throughout treatment. The probability of central venous port removal in children with solid tumors throughout the follow-up period was significantly lower, at 16% in Wilms tumor, 19% in brain tumor, and 14% in neuroblastoma. The Kaplan-Meier method was also used to analyze the reliability (length of function) of the venous port. The predicted average survival (functioning) time of the venous port was 1512 days (4 years) (**Figure 4**).

When performing the analysis of the venous port’s function, factors that may affect the system’s operating time were also taken into account. The following factors were shown to affect the duration of the port: the presence of a hematoma at the implantation site, the type of venous port implanted, and the occurrence of infection.

**Figure 5:**
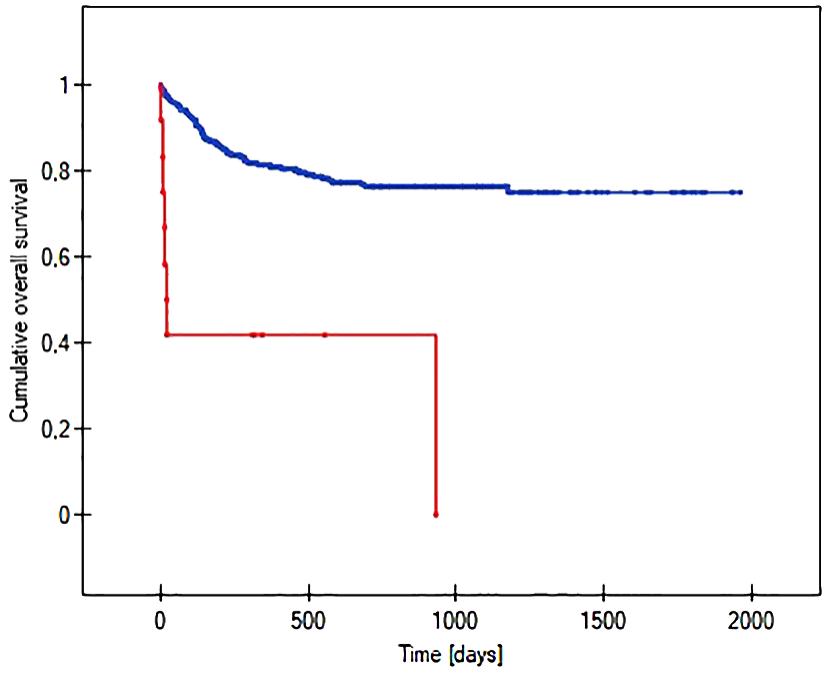
Graph showing the comparative analysis of the length of the vascular port function in relation to the formation of a port pocket hematoma after implantation. The red line indicates the presence of a port pocket hematoma, and the blue line indicates its absence (p=0.0296).

**Figure 6:**
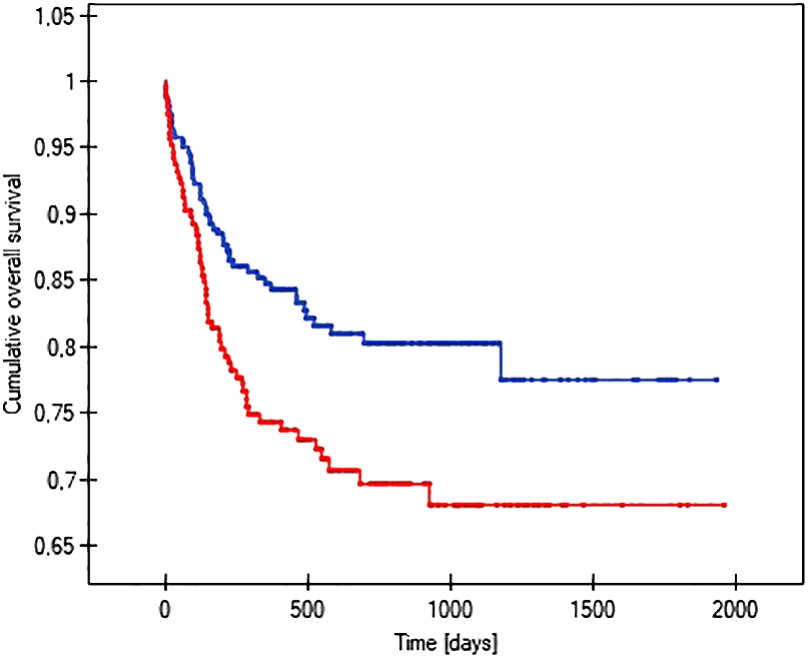
Graph showing the comparative analysis of the length of vascular port function in relation to the type of port implanted. The red line indicates the polyurethane catheter, and the blue line indicates a silicone catheter (p=0.0365).

**Figure 7:**
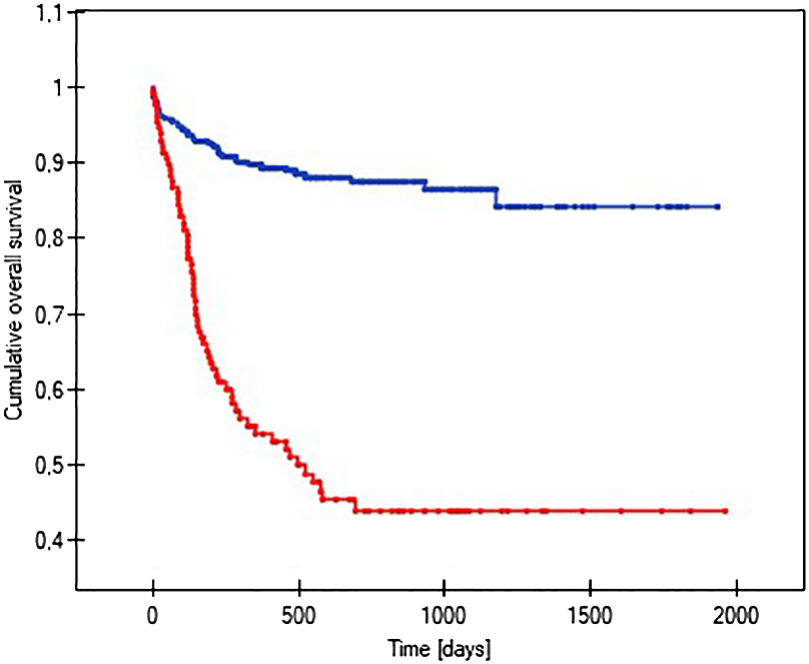
Graph showing the analysis of the length of function of infected and non-infected vascular ports. The red line indicates the infection of the port, and the blue line indicates the absence of infection (p=0.047).

The probability of earlier venous port loss in children who developed a hematoma at the implantation site was more than 6 times higher than in patients without this complication (p=0.0296) (**Figure 5**). We found that the probability of earlier venous port loss was 1.7 times higher with a polyurethane catheter than with a silicone catheter (p=0.0365) (**Figure 6**). We also proved that the probability of earlier venous port loss in children who developed a venous port infection was more than 5 times higher than in patients without this complication (p=0.047) (**Figure 7**).

## Discussion

Central venous ports have become a standard in providing safe, long-term chemotherapy in adult and pediatric oncology patients, offering patient comfort, ease of medical use, system longevity and advantage over other long-term venous accesses (8, 9, 18). According to studies, in addition to the widespread comfort of the port, an advantage of the system is the lower incidence of infection compared to other catheters used in long-term treatment (10, 16–20). Aseptic implantation techniques are critical for minimizing early complications like infection. Additionally, the surgical team’s expertise is paramount in preventing early complications such as pneumothorax, arterial puncture, or operative site hematomas. The maintenance and care of the venous port system, particularly in preparation and blood collection, are vital, necessitating a skilled nursing staff trained in strict aseptic protocols (21).

The prevalence of port use varies considerably across centers, with some adult patient populations showing rates as low as a few percent annually to about 15% of those receiving chemotherapy (11, 18). In our Department, venous ports are a standard for all children receiving chemotherapy, emphasizing the importance of comfort and secure administration routes for long-term treatments. The common practice of venflon-type peripheral intravenous catheterization, often painful for children, highlights the benefits of venous ports (22, 23). Specific patient populations, such as those with chronic conditions like hemophilia, cystic fibrosis, mucopolysaccharidosis, and certain chronic neurological disorders, including drug-resistant epilepsy, necessitate individualized port implantation strategies. In severe hemophilia, where routine clotting factor administration is critical, the use of a venous port has proven effective for both regular and emergency treatments. Ljung et al.’s study in a pediatric hemophilia cohort demonstrated a 70% success rate free of complications, with a 30% infection rate among those with implanted ports, reflecting caregiver satisfaction (24). In cases of cystic fibrosis, where frequent hospitalizations for treatment are necessary, port implantation improved the quality of care without complications, pleasing patients, families, and treating physicians. Similarly, for patients with immunodeficiencies requiring recurrent transfusions or those with congenital syndromes necessitating frequent hospital stays, port implantation served as a justified and beneficial intervention.

In our Department venous ports were placed in 12 patients with the severe form of hemophilia A. In 2 children the procedure was complicated with the formation of a huge hematoma of the operated area, despite adequate pharmacological preparation of the patient for the procedure, resulting in subsequent loss of the system. Laboratory tests showed that patients who experienced postoperative bleeding developed neutralizing antibodies against factor VIII. In our study, ports were implanted in 4 patients with cystic fibrosis. No complications were observed after port implantation in these patients, achieving complete satisfaction of patients, their parents, and pulmonologists. In 9 patients the indication for port insertion was immunodeficiency, causing the need for frequent transfusions of immune antibodies (e.g., every 1-2 months). Syndrome of congenital anomalies was indication for venous port in 11 patients.

The literature confirms the widespread yet safe use of an implantable port-type intravenous system in pediatric patients requiring long-term treatment (4–7, 24–27). The Seldinger method is described as a safe technique that allows the central vessel to be reached with relative ease (28). The literature indicates that the experience of the operator using the technique plays a significant role. Dheer et al. observed that complications associated with the method were observed less frequently with greater familiarity with the procedure by the person performing it. They emphasized that it is a safe technique, provided it is performed by an experienced operator (12). Our center’s experience aligns with literature findings, highlighting the essential role of comprehensive medical personnel training. The frequency of early complications in the pediatric population occurring in the Poznan center was similar to international literature data for both adult and pediatric patients (5, 29–34). The incidence of early complications, particularly pneumothorax, correlated with the operator’s experience, being higher among physicians in training. This underscores the necessity for careful postoperative monitoring, as radiological evidence of pneumothorax may not be immediately apparent, and children may not verbalize symptoms promptly. Available publications indicate that pneumothorax did not require surgical management every time. In the case of dyspnea, drop in saturation, and emphysema increasing on a follow-up radiograph, it was necessary to place a pleural drain (16). In a publication by Barbetakis et al. in a group of 700 adult cancer patients, pneumothorax occurred in 2.2% of cases (35).

Intravenous port implantation is associated with typical complications of central venous cannulation. The literature shows many of the determinants for these complications (12), primarily derived from adult patient analyses. Early complications following subclavian vein access for port implantation often include arterial puncture, potentially leading to subcutaneous hemorrhage, pleural effusion, or, in severe cases, airway constriction. A substantial adult cohort (n=700) exhibited a hematoma incidence of 2.2% and arterial puncture at 1.6% (35). Moreover, studies have reported cardiac arrhythmias in 2.1% of cases, and infrequently, issues like catheter kinking, damage to the port chamber, and locus infections (35). Research by Shankar et al. into a pediatric demographic (n=122) noted similar early complications within three weeks post-implantation, such as hematomas, catheter tip displacement, port obstruction, and infections (30). Our findings in 474 implantations corroborate these figures, with pneumothorax (3%) necessitating pleural drainage in a majority (80%) and hematomas (2.5%) at the implantation site. There was no observed correlation between these complications and patient age. Notably, hematomas significantly increased the risk of port loss, underscoring the importance of effective perioperative management and potentially necessitating system removal when conservative measures fail.

Late complications, estimated at roughly 6%, predominantly manifest as infections or thrombosis at the catheter’s distal end (16). These complications, whether within the port tunnel, pocket, or in relation to the catheter itself, bacteremia and septic thrombophlebitis, were documented in 6.2% of the pediatric subjects studied by Shankar et al. (30). Our investigation revealed a higher incidence, with early infections occurring in 3% and late infections in 24% of cases. In the available literature, the percentage of port infections is observed in the range of 2.2%-9% (36, 37). This discrepancy can be largely attributed to the high proportion of leukemia and lymphoma cases within our cohort, where aggressive chemotherapy regimens contribute to sustained immunosuppression conducive to infections. Our analysis also highlighted an increased infection susceptibility in patients with acute lymphoblastic leukemia, acute myeloid leukemia, non-Hodgkin’s lymphoma, and neuroblastoma, findings that align with other literature (1, 16–18, 34).

Pathogens responsible for these infections have evolved in parallel with shifts in antibiotic usage and the materials used in catheters and ports. Most infections originate from skin-dwelling bacteria or contaminants from healthcare providers’ hands. The predominant pathogens are skin bacteria such as *Staphylococcus epidermidis, Klebsiella* spp.*, Acinetobacter* spp., and *Candida* spp. fungi. Infection rates correlate with the composition of the port, its placement, and the patient’s overall health. According to Bagnall-Reeb et al., the typical duration for antibiotic therapy treating port infections spans 7 to 21 days, with success rates between 60 and 91% (38). Our center’s data are consistent with these figures, with *Staphylococcus epidermidis* being the most frequent infective agent. Approximately half of the patients with infected ports responded positively to a median 14-day antibiotic regimen, yet over half necessitated port removal due to infection. In our cohort, 14% of children experienced port loss due to infection, leading to recommendations for prophylactic antibiotics in immunocompromised individuals (18, 39, 40).

Catheter-related thrombotic obstruction rates have been reported between 27-66% in adults and 7-50% in pediatric patients (39). This complication is often intertwined with infection, as the fibrin sheath encasing the catheter creates a conducive environment for microbial growth (10). In our study, significant thrombosis occurred in 0.8% of cases, often associated with genetically predisposed thrombophilia or cancer types prone to thrombosis. Unlike the adult population, where anticoagulant prophylaxis is commonplace, pediatric practice has not shown a significant reduction in thrombosis incidence with such treatment (39).

Furthermore, rarer complications include catheter or port membrane damage, causing leakage, venous displacement of the catheter, or port obstruction not amenable to pharmacological intervention, necessitating surgical revision. These complications represented less than 3% in our series.

A notable association was identified between the type of venous port used and the likelihood of port loss. Early port failure was 1.7 times more probable with polyurethane catheters compared to silicone. Moritz Wildgruber et al. observed a higher prevalence of infections and obstructions in polyurethane catheter patients (41). However, to demonstrate the effect of the venous catheter material on the incidence of infections and thus port loss, we would need an analysis performed on a group of patients comparable in terms of underlying disease and the use of a venous catheter of the same diameter constructed from different materials. These assumptions could not be met in the study group, as the venous catheters differed in diameter, and the study groups (younger and older children) showed a discrepancy in terms of the distribution of the underlying disease.

The main limitations of our study are the retrospective nature of the study and the single-center design. Factors not taken into account in the study design, which may have influenced the results include postoperative port care and its accuracy by the nursing team. Despite general standards, any improper handling of port use may affect the rate of infection.

The high incidence of late infectious complications in our center calls for further analysis and elimination of factors that may influence the occurrence of port infections. Another limitation of the study may be the population we studied, primarily oncological patients, who made up the majority of the group. These are particularly vulnerable patients and the results we obtained may not be applicable to other populations. Although we presented a significant group of patients, our study was conducted using data from a selected period of only 8 years.

Despite the limitations listed, our research has its strengths, which include among others, the fact that it is based on a large number of patients and that the data was collected in an extremely thorough manner (42–44).

## Conclusions

TIVADs provides an effective and reliable system for long-term intravenous treatment in pediatric patients, catering not only to oncological therapies but also to a spectrum of chronic conditions. Employing the Seldinger technique, subclavian port insertion is established as a swift, minimally invasive, and generally safe practice.

The type of port, including the material of the port’s venous catheter, and the diagnosis of the underlying disease influence the durability of implantable intravenous systems. Surgeon’s experience is related to the incidence of complications associated with the procedure.

Addressing the elevated rates of late-stage infections observed in our cohort necessitates a meticulous reevaluation of current protocols and practices, aiming to mitigate risk factors and enhance patient outcomes.

## Data availability statement

The raw data supporting the conclusions of this article will be made available by the authors, without undue reservation.

## Ethics statement

The studies involving humans were approved by the Bioethics Committee at the Karol Marcinkowski Medical University in Poznan. The studies were conducted in accordance with the local legislation and institutional requirements. Written informed consent for participation in this study was provided by the participants’ legal guardians/next of kin. Written informed consent was obtained from the individual(s), and minor(s)’ legal guardian/next of kin, for the publication of any potentially identifiable images or data included in this article.

## Author contributions

PS-S: Conceptualization, Data curation, Formal analysis, Funding acquisition, Investigation, Methodology, Project administration, Supervision, Visualization, Writing – original draft, Resources. SM: Conceptualization, Data curation, Formal analysis, Funding acquisition, Investigation, Methodology, Visualization, Writing – original draft. DJ-L: Conceptualization, Project administration, Software, Supervision, Visualization, Writing – review & editing, Formal analysis, Funding acquisition. KMi: Conceptualization, Data curation, Funding acquisition, Investigation, Methodology, Writing – review & editing. KMa: Conceptualization, Data curation, Funding acquisition, Methodology, Project administration, Writing – review & editing. AK: Conceptualization, Data curation, Funding acquisition, Investigation, Validation, Writing – review & editing. DZ: Data curation, Formal analysis, Funding acquisition, Writing – review & editing, Investigation, Software. PM: Conceptualization, Funding acquisition, Methodology, Project administration, Supervision, Visualization, Writing – review & editing.
